# Postural demands modulate tactile perception in the lower limb in young and older adults

**DOI:** 10.1038/s41598-025-06736-w

**Published:** 2025-06-20

**Authors:** Fabian Dominik Wachsmann, Katja Fiehler, Dimitris Voudouris

**Affiliations:** Experimental Psychology, Justus Liebig University, 35394 Giessen, Germany

**Keywords:** Balance control, Posture, Tactile modulation, Aging, Human behaviour, Perception, Motor control, Sensory processing, Somatosensory system, Cognitive ageing

## Abstract

Balance control requires the continuous integration of feedback signals from several sensory organs with feedforward estimates about the state of the body. Such feedback signals are important for standing upright, as shown in increased and more variable sway patterns when sensory feedback is compromised, for instance when standing with eyes closed or on unstable surfaces that make cutaneous signals from the foot less reliable. Poorer sensory processing is also considered to arise during healthy aging due to a decrease of the reliability and transmission rate of feedback signals. Here, we are interested in how processing of tactile signals from the lower leg is modulated when balance control is challenged and how this interacts with age-related sensorimotor changes. We examined tactile sensitivity on the lower leg during sitting, standing on stable ground, and standing on unstable ground (foam). We quantified the center of pressure during the two standing conditions by determining the area of a 95% confidence interval ellipse as well as the total displacement of the center of pressure. Tactile sensitivity was assessed by asking participants to detect brief vibrotactile probes of various intensities to the lower leg. As expected, postural sway increased when standing on foam than stable ground for both age groups. When postural demands were minimal (sitting), tactile sensitivity was overall poorer in older than younger adults. Tactile perception was also poorer when standing on foam than on the stable ground, for both age groups. We conclude that increased postural demands reduce reliance on tactile signals from the lower limb in both young and older adults.

## Introduction

Standing upright requires a sophisticated interplay between feedback and feedforward processes. Humans estimate the current state of their body stance by sampling and synthesizing visual, vestibular, proprioceptive, and tactile signals based on their reliability^1,2^ and integrate this sensory information with previous experiences about the prevailing dynamics to generate descending motor commands and efference copies, through which they can establish sensorimotor predictions about future sensory states^3^. This process requires access to the most recent sensory input, which can facilitate postural adjustments to retain upright stance.

Poorer sensory input can lead to compromised postural control^2^. For example, when people stand with eyes closed, their centre of pressure (COP) can be subject to larger and more variable displacements^4^ indicating postural instability. Besides vision, somatosensation plays a central role in keeping upright. For example, lightly touching an object with the upper-limb can reduce postural sway, even when the contact forces do not offer sufficient physical support^5,6^. Such light touch provides tactile and proprioceptive signals about arm position, which can inform the postural system about necessary adjustments, and even reduce the impact of hampered sensory input from other modalities that might have a destabilizing effect on posture^7^. In addition to light touch, cutaneous signals from the lower limb have a key role in postural control. Obviously, cutaneous afferents from the foot sole provide key information about the state of the upright body^8^ so when cutaneous input from the foot sole is degraded, such as when standing on surfaces of low compliance (e.g., foam), postural sway increases^9–11^. Although the foot sole is the primary somatosensory access point to obtain information about the relationship between the standing body and the ground, cutaneous signals also from other parts of their lower limb can contribute to postural control. For example, light pressure arising at the lower leg can reduce postural sway, and the contribution of these input signals is even more pronounced when they arise further away from the foot (e.g., at the knee and calf compared to the ankle;^12^). Moreover, cutaneous afferents from the lower leg encode information about the direction of ankle movements, and this information matches well the directional information conveyed by the lower leg muscles^13^. This further highlights the central role of tactile feedback signals from the lower leg to estimate the sensory state of the limb, and to control whole-body posture.

Despite the importance of tactile signals for movement control, tactile sensitivity is often compromised in a limb that is about to move or is already in motion. Voluntary movements can suppress tactile signals arising from the moving limb, a phenomenon known as *tactile suppression*. This suppression primarily stems from central efference copy mechanisms^14,15^ which predict the sensory states of the moving limb and downweigh associated feedback. However, peripheral processes related to movement, such as proprioceptive afferents generated by the action itself, may also suppress tactile stimuli on the moving limb, likely through masking. For instance, suppression is evident also during passive movements^16–18^ where the involvement of descending motor commands is unlikely. Importantly, tactile suppression is not an all-or-nothing mechanism but is modulated by task demands. For example, suppression weakens, disappears, or even reverses to tactile enhancement when somatosensory feedback from the probed limb is critical for the task. As a matter of fact, the strength of tactile suppression during sensory-guided reaching or grasping is temporally modulated, as suppression can diminish at critical moments^19,20^. Similarly, while standing, tactile sensitivity from the lower leg increases shortly before a visual perturbation that could destabilize balance^21^. Interestingly, tactile afferents from a stationary leg can also be enhanced shortly before the other leg initiates a step, as the stimulated leg becomes important for supporting whole-body balance^22^. Additionally, afferents from the ankle joint are enhanced just before the stimulated leg touches the ground during locomotion^23^. Together, these findings suggest that afferent signals from various body parts are processed dynamically, depending on their relevance to the task at hand.

Sensory processing is typically poorer in healthy aging^24,25^ while sensorimotor functions are slower as humans grow older^25,26^. Such compromised sensorimotor processes may explain why some studies demonstrate stronger reliance on predictive control during aging. Accordingly, tactile suppression increases in older adults^24,27^,but see also^28^ which might reflect a compensatory mechanism to adapt to the poorer sensorimotor functionality. However, other studies show poorer predictive control in older than younger adults, as reflected in smaller and delayed anticipatory postural adjustments before self-imposed perturbations^29^. So far, it is unknown how processing of tactile information from the lower leg, which is essential for maintaining upright balance, is modulated by postural demands and their associated motor responses in healthy young and older adults.

Here, we examined the modulation of tactile signals from the lower leg when participants were either sitting, standing on stable ground, or standing on an unstable (foam) surface. We expected increased postural sway when standing on an unstable than stable surface in both age groups. Based on previous work^24,25^ we further expected poorer tactile sensitivity at the lower leg during sitting, when no postural demands are introduced, in older compared to younger adults. If standing on an unstable surface requires increased reliance on feedback signals from the lower leg, tactile sensitivity on the lower leg might be enhanced when standing on foam than on stable ground. Alternatively, if standing on an unstable surface introduces increased afferent inflow due to muscle stiffness or pronounced sway, tactile suppression might be stronger when standing on foam than on stable ground. If aging influences tactile suppression depending on the postural demands, there should be an interaction.

## Methods

### Participants

We recruited 23 young (24.04 ± 4.25 years old, range: 18–35; height: 171.26 ± 10.72 cm; 17♀, 6♂) and 20 older (64.10 ± 5.09 years old, range: 55–72; height: 170.15 ± 10.76 cm; 15♀, 5♂) healthy participants. They were free from any known neurological or musculoskeletal issues at the moment of the experiment that could hinder their participation, and they had normal or corrected-to-normal vision. Younger participants were recruited from student pools of the Justus Liebig University Giessen. Older participants were community-dwelling and were recruited through personal contacts of the authors and through internal mailing lists of the Justus Liebig University Giessen. All participants or their legal guardians signed an informed consent. The study was approved by the local ethics committee of the Justus Liebig University Giessen. All methods were carried out in accordance with the “World Medical Association Declaration of Helsinki” (2013, except for § 35, pre-registration)^30^. At the end of the experiment, participants received either 8€/hour or course credits.

## Apparatus

A custom-made vibrotactile stimulation device (Engineer Acoustics Inc., Florida, US), which will be referred to as a “tactor”, was attached to the skin over the *musculus gastrocnemius lateralis* of the participant’s right calf, a muscle primarily used for balance control^31^. This tactor consists of a small housing (22 × 45 × 5 mm) and a round actuator (6 mm in diameter) that can generate vibrotactile stimuli of fine-grained duration, frequency and amplitude. The tactor was fixed at 3 cm medial of 2/3 of an imaginary line connecting the heel with the *caput fibulae.* The tactile device was securely positioned in that specific spot and delivered probe tactile stimuli of various intensities that act as a proxy for tactile processing during upright stance. This particular body part was chosen based on previous studies showing that tactile input from the lower leg plays a crucial role in postural control^12,21^. Unlike the foot sole or Achilles’ tendon, this area experiences minimal skin deformation, which is a known confounding factor in tactile perception^32^. Additional tape was used to fix loose wires or clothing to the participant’s body so that they would not cause sensations that could be misinterpreted as vibrations or interfere with task performance. To manipulate the postural demands and their associated motor responses, we asked participants to either sit, stand on a stable surface, or stand on a piece of foam, similarly to previous work^11,33^. We used a force plate (AccuSway, AMTI, Massachusetts, US) to sample the (COP) at 300 Hz to assess postural behaviour in the two standing conditions only. In the sitting condition, participants sat on a chair with their feet on the force plate and their knees at ∼90 deg. In the standing condition, they stood directly on the force plate (∼5 cm from the ground), while in the foam condition participants stood on a balance pad (50 × 40 × 6 cm, density of 81.3 kg/m^3^; SISSEL BalanceFit Pad, novacare GmbH, Bad Dürkheim, Germany) that was placed on top of the force plate. Participants had their feet at shoulder-width apart in all three conditions. They could also choose to be either barefoot or with their socks on, and were required to retain this choice throughout the experiment. To ensure a comfortable and safe environment, an additional experimenter stood beside the participant to reduce any possible fear of falling by providing support in the unlikely event of a fall (no participant ever fell). For the standing conditions, participants were upright with their arms relaxed at their sides and had to fixate a circular point (18 cm diameter) on the facing wall, ~ 230 cm in front of them and at a height of 185 cm.

## Procedure

Data acquisition as well as pre-processing and analysis were handled by custom-made Matlab R2021a software (The Mathworks, Massachusetts, US).

The experiment consisted of 5 phases (familiarization, training, and three experimental blocks). During familiarization, we presented 5 trials with a vibrotactile probe (50 ms, 250 Hz) to the participant’s lower leg while standing, ranging from low (peak-to-peak amplitude: 31.6 μm) to strong (peak-to-peak amplitude: 284.4 μm) intensities. This familiarization phase allowed participants to understand how the stimuli felt. Please note that this vibration is irrelevant for movement control and is used as a proxy to probe tactile perception on a body part that is relevant for the performed task, similarly to the procedure used to probe tactile suppression during arm and finger movements e.g.^14,34^. In addition, the intensity and duration of those probe vibrations are much different from those known to cause illusory postural sensations through the activation of Golgi tendon organs and muscle spindles e.g.^35,36^.

A training phase that included 10 trials was presented after the familiarization procedure. Participants now stood on the force plate. Each trial started with the experimenter pressing a button that triggered data collection and within a variable interval of 2–3 s after that moment, a vibrotactile probing stimulus (50 ms, 250 Hz) of various intensities was presented at their lower leg. An auditory cue indicated the end of the trial right after the vibration and prompted participants to respond as to whether they had noticed a tactile stimulus, if they had not responded already. An experimenter logged the responses to the host PC via button presses. The stimulus intensity for each trial was determined using a QUEST algorithm (Psychtoolbox Version 3.0.18), which employs a Bayesian approach to estimate psychometric function parameters using prior knowledge. We used a Weibull function with β = 3.5, as recommended for two-alternative forced choice tasks^37^ while the prior mean and standard deviation were estimated from a pilot study with young participants standing with their feet together (*N* = 16; exposed to 50 stimuli of varying intensities). We conducted the training phase to ensure participants fully understood the task and could accurately identify the relevant tactile probes. This step was crucial, as the quality of initial trials in a QUEST staircase method directly influences the determination of the intensities of subsequent trials.

**Fig. 1:**
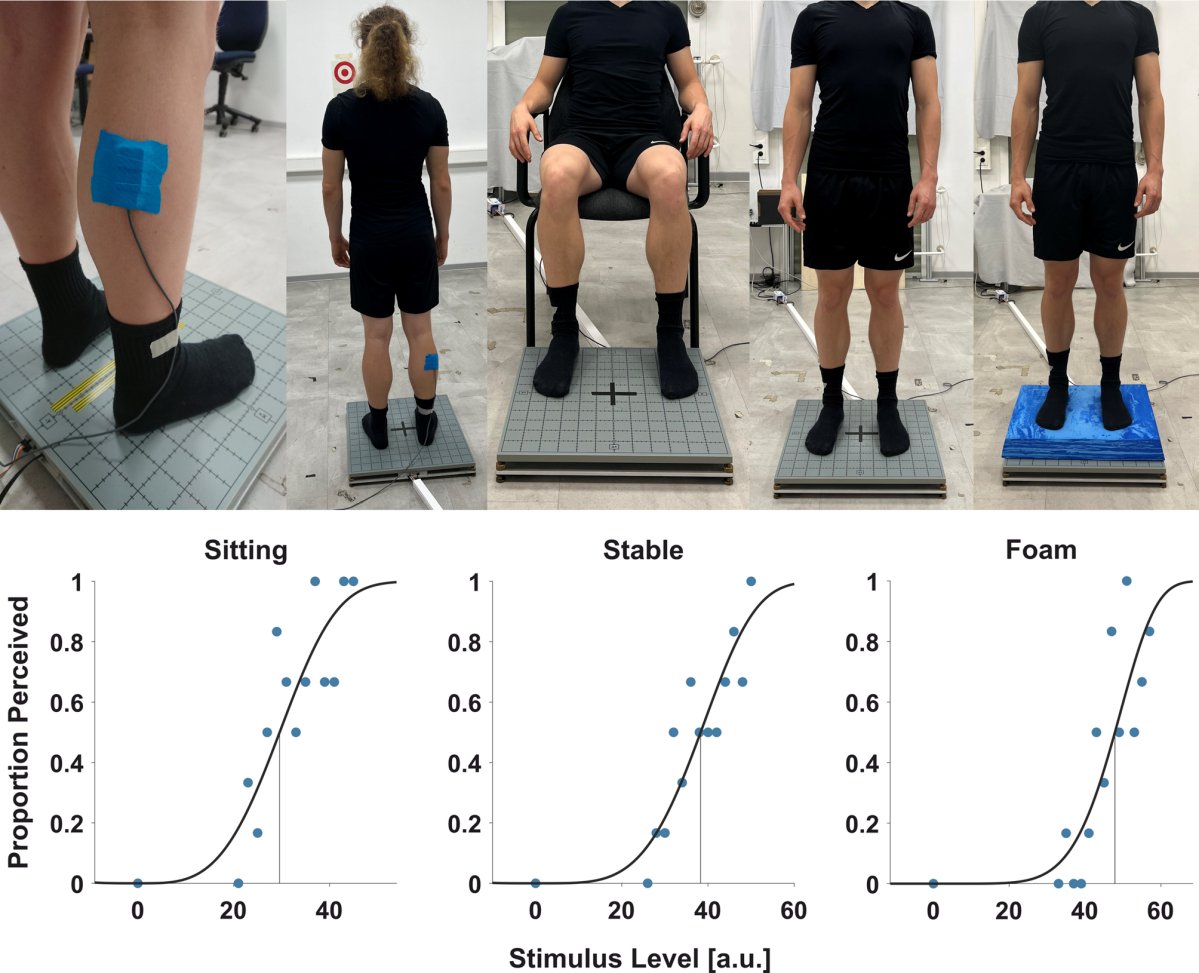
**Experimental setup and psychometric functions.** From left to right in the upper panel, pictures of the tactor placement, the fixation location (red circle), the sitting, the stable standing, and the unstable standing condition. In the lower panel there are 3 exemplary psychometric functions for the three conditions from the first participant. Blue dots represent the ratio with which stimuli of different intensities were perceived. The units are arbitrary. The vertical line indicates the estimated detection threshold as the 50% level.

This training phase was followed by the 3 experimental blocks, each consisting of a single condition (sitting, stable, foam; Fig. 1), presented in random order. Each experimental block was preceded by a calibration phase consisting of 20 trials, during which the intensities of the tactile probes were recalibrated using a QUEST algorithm with parameters identical to those previously described. The reason for doing so was not only the fact that individual detection thresholds are considerably variable across participants e.g.^38^ and differ between age-groups e.g.^24^, but that they may also differ under various postural demands. Therefore, we aimed to determine a suitable probing intensity range for each participant and for the underlying postural condition. Once these 20 trials were completed, we used the mean of the estimated probability density function to construct a range of probing stimuli for the respective participant and postural condition. This allowed us to tailor our probing stimulus range around each participant’s detection threshold and therefore to minimize the risk of ceiling or floor effects. We used this range of stimuli to assess tactile perception with the method of constant stimuli during the experimental blocks. The range was constructed by choosing 6 stimulus intensities that were weaker and another 6 intensities that were stronger than the threshold estimated by the QUEST, resulting in a total of 13 intensities (including the QUEST threshold) that were separated by a peak-to-peak displacement of the actuator equal to 6.32 μm. Sometimes the estimated QUEST threshold was so low that it was not feasible to choose 6 unique intensities below the QUEST threshold that were both positive and non-zero. In such cases (17 blocks in 11 participants) every intensity level that would correspond to an intensity of 0 or lower was handled as a level without stimulation (catch trial). The resulting range of presented stimuli can therefore be different for every condition and participant to optimize psychometric probing and fitting. We presented 6 trials for each stimulus level that had an intensity larger than 0, and another 22 mandatory catch (no-stimulation) trials resulting in a total of 100 trials per postural condition. The intensities of the probing stimuli were presented in a random order within each block. While participants were allowed to take breaks and sit at a chair next to the force plate whenever they wanted, mandatory breaks of at least one minute were enforced during both standing conditions after 20 QUEST trials and after every 25th trial. As in the training phase, the tactile probe was presented within 2–3 s after trial onset and, again, the end of a trial was indicated by an auditory cue.

Each trial lasted ~ 5 s, including the time window for responding to the tactile stimulus. This might appear short for assessing postural behavior, as most studies examine body balance in longer time intervals (e.g., 30 s). However, our main interest is in assessing tactile sensitivity, which requires several trials. In our experiment we presented more than 300 trials per participant, and so we decided to keep the duration of each trial short to facilitate the experimental procedure, especially for our older adults.

### Data analysis

**Fig. 2:**
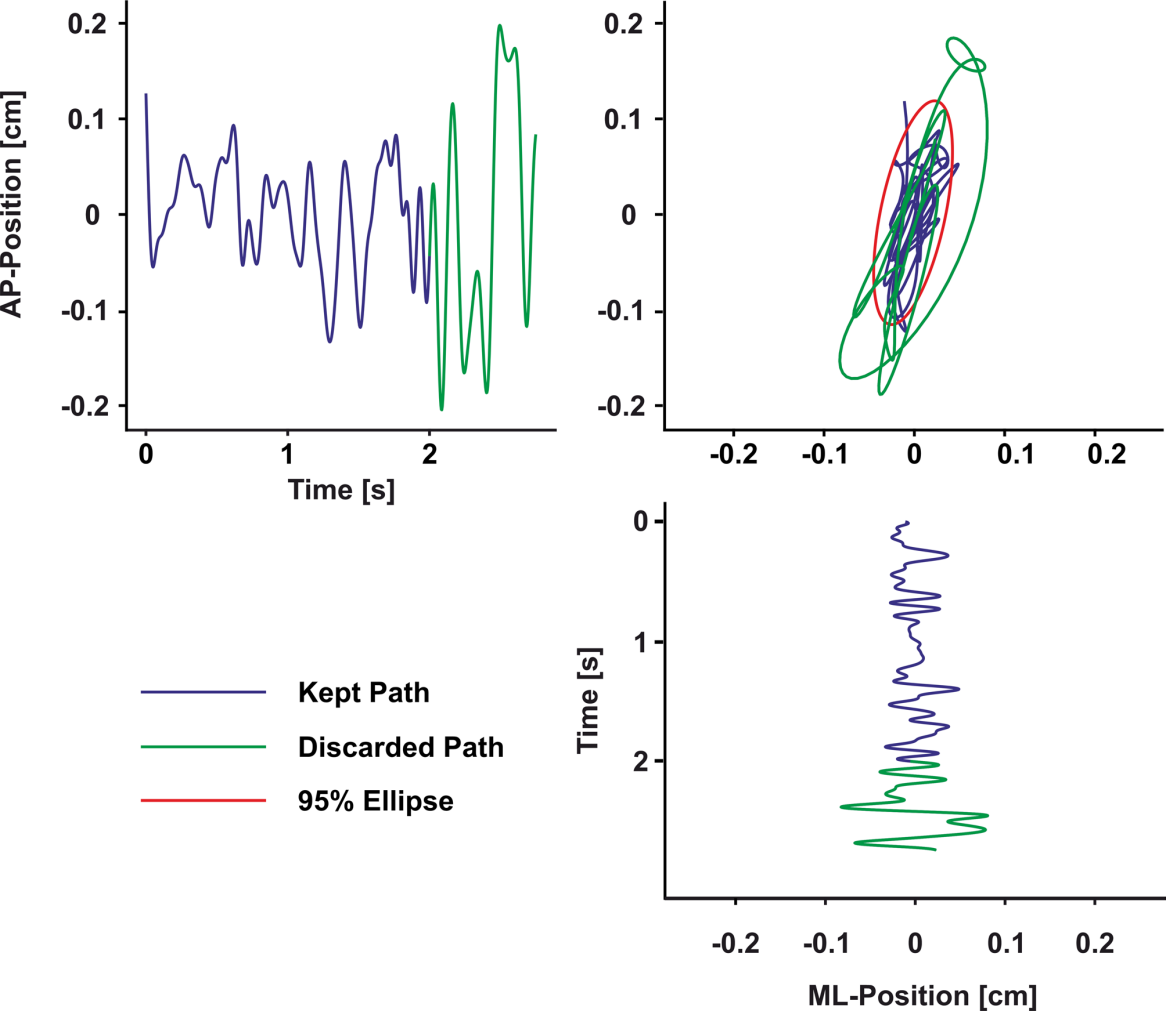
**Kinetic data preparation.** The panels show the statokinesiogram of the anterior-posterior position (top-left) and the medio-lateral position (bottom-right) of the center of pressure within one trial. On the top-right is the stabilogram of that same trial. Blue lines indicate the 2 s truncated data that went into the analysis, while the green lines are the discarded rest of the trial. The red ellipse corresponds to the sway area analysis.

To evaluate whether upright stance changed between the two standing conditions, we analyzed COP characteristics. We first pre-processed the COP data by subtracting the mean mediolateral and anteroposterior values from each trial’s time-series data. We then smoothed the data using a zero-lag, 4th-order Butterworth low-pass filter with a 10 Hz cutoff. Since the tactile probe was presented at the earliest within 2 s after trial onset, which was critical to keep the duration of the experiment within reasonable limits, we truncated our COP data to include the samples obtained during the first 2 s of each trial, eventually excluding any possible influence of the tactile stimulation itself and of the verbal response to this tactile stimulus on posture (see Fig. 2). It is, however, important to note that participants stood on the force plate for the whole block of 100 trials and the first trial started after a brief familiarization phase, so any non-stationary parts that appear in the beginning of standing task are reduced^39^. For each trial we calculated the area of a 95% confidence interval ellipse of the COP data, further called *sway area*, to quantify stability in the spatial domain. We also calculated the total two-dimensional displacement of the COP path, further called *sway length*, to quantify stability more in the temporal domain. We then averaged these values across all trials per condition of each participant.

To evaluate how perception of tactile signals is influenced by the underlying postural conditions and for each age group, we fitted the responses to tactile stimuli in each of the three postural conditions, separately for each participant, with a Weibull function using the Psignifit 4 toolbox^40^ in Matlab. We defined the tactile detection threshold as the stimulus intensity at a 50% detection rate, adjusting for lapse and guess rates. The slope at this threshold served as a measure of perceptual sensitivity. To assess tactile modulation during standing while also accounting for individual differences in tactile perception during rest (sitting baseline), we normalized each participant’s detection thresholds and slopes by subtracting their sitting baseline values from those obtained in the two standing conditions, resulting in two normalized threshold and slope values per participant (Δthreshold and Δslope).

Three young participants were excluded from all kinetic analyses due to a technical malfunction of the force plate, leaving us with a sample of 20 younger and 20 older adults with full data sets. We were first interested in whether tactile sensitivity changes as a function of age. We decided to focus on tactile sensitivity only during sitting as this provided us with pure age-related sensory changes in tactile sensitivity, taking out any possible influences of posture itself. For instance, when standing, there may be differences in skin stretch or muscle activity at the lower leg, which can influence on their own tactile sensitivity^32^. In addition, performing a tactile detection task while maintaining balance or not could impair the simultaneously performed perceptual task^41^ which could have influenced tactile sensitivity across participants and age groups.

For the analysis, we first confirmed that the false alarm rates during the sitting baseline were smaller than 0.2. In a second step, we excluded participants from the baseline analysis if their estimated baseline thresholds were beyond the strongest intensity presented in that block (1 younger and 2 older adults). Consequently, these three participants were also excluded from the Δthreshold and Δslope analyses because these two analyses require reliable sitting baseline values. In addition, participants with a false alarm rate above 0.2 or estimated thresholds beyond the stimulation range in at least one standing condition were excluded from the Δthreshold and Δslope analyses (3 young, 1 old). In a final step, we excluded participants as outliers if their datapoints were outside of a 2 interquartile range. This was done separately for the baseline and the Δthreshold/Δslope analyses. It resulted in additionally excluding 2 young adults from the baseline thresholds, 2 young and 2 old adults for the baseline slopes, 1 young and 1 old adult for the Δthresholds, and 4 young and 7 old adults for the Δslopes.

For the kinetics, we excluded participants as outliers based on the interquartile ranges of each of the two variables. Specifically, we excluded only 2 old participants based on their sway area and another 2 old participants based on their sway length values. No young adult was excluded from the kinetic analyses.

### Statistical analysis

To examine if postural demands affect upright stance in young and older adults, we conducted two separate 2 (age groups) x 2 (postural demands) repeated measures mixed ANOVAs on sway area and sway length (38 datasets). To confirm that aging reduces overall tactile perception, as has been shown before^25^ we compared detection thresholds (38 datasets) and slopes (36 datasets) during the sitting baseline between age groups using one-sided independent t-tests. To examine the modulation of tactile sensitivity as a function of postural demands and aging, we conducted two separate 2 × 2 repeated measures mixed ANOVAs with the factor’s postural demands (stable, foam) and age (young, old) on the normalized detection thresholds (34 datasets) and slopes (26 datasets). We set our alpha to 0.05, and we report effect sizes as *η*_*part*_^2^ following the calculations and recommendations by Corell^42^. Statistical analyses were conducted in JASP version 0.18.1 (University of Amsterdam, The Netherlands).

## Results

### Kinetics

**Fig. 3:**
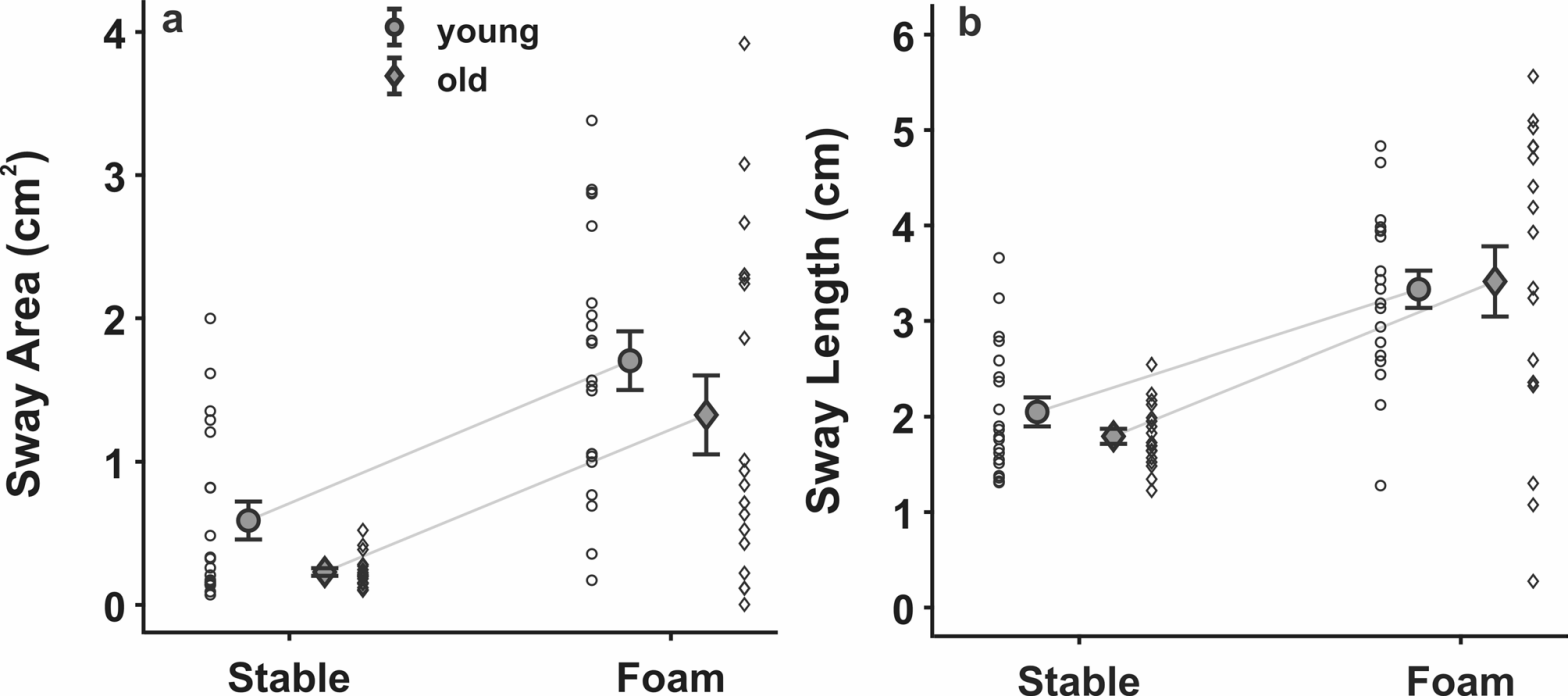
**Kinetic modulation.** Comparison between postural demands and age group for (**a**) sway area, as indicated by the 95% Confidence Interval of the COP, and (**b**) sway length, as indicated by the total length of the COP. Means and single subject data for the young (circles) and older adults (diamonds) are depicted with standard error as error bars.

As expected, sway area and sway length were higher when standing on foam than on the stable surface (area: *F*_*1,36*_ = 47.492, *p* < 0.001, *η*_*part*_^*2*^ = 0.569; length: *F*_*1,63*_ = 54.624, *p* < 0.001, *η*_*part*_^*2*^ = 0.603; Fig. 3a-b), confirming that our experimental manipulation led to the expected increase in postural sway. However, sway area did not differ between age groups *(F*_*1,36*_ = 3.274, *p* = 0.079, *η*_*part*_^*2*^ = 0.083) and there was no interaction *(F*_*1,36*_ = 0.003, *p* = 0.956, *η*_*part*_^*2*^ < 0.001). Sway length was also not influenced by aging (*F*_*1,36*_ = 0.128, *p* = 0.723, *η*_*part*_^*2*^ = 0.004) and there was no interaction either (*F*_*1,36*_ = 0.741, *p* = 0.395, *η*_*part*_^*2*^ = 0.020).

## Tactile perception

**Fig. 4:**
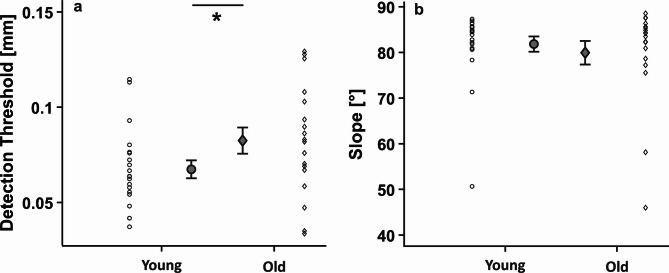
**Age effects on tactile perception during sitting.** Comparison between the age groups for (**a**) detection thresholds, and (**b**) slope of the psychometric function in the baseline, sitting condition. Means and single subject data for the young (circles) and older adults (diamonds) are depicted with standard error as error bars. * *p* < 0.05.

Older adults had overall higher detection thresholds than young adults during sitting (*t*_*36*_ = 1.843, *p* = 0.037, *η*^*2*^ = 0.082; Fig. 4a), in line with previous findings showing poorer tactile sensitivity in aging^24^. However, there were no statistically significant differences in the slopes of the baseline psychometric functions between the two age groups (*t*_*34*_ = 0.482, *p* = 0.683, *η*_*part*_^*2*^ *=* 0.007; Fig. 4b).

**Fig. 5:**
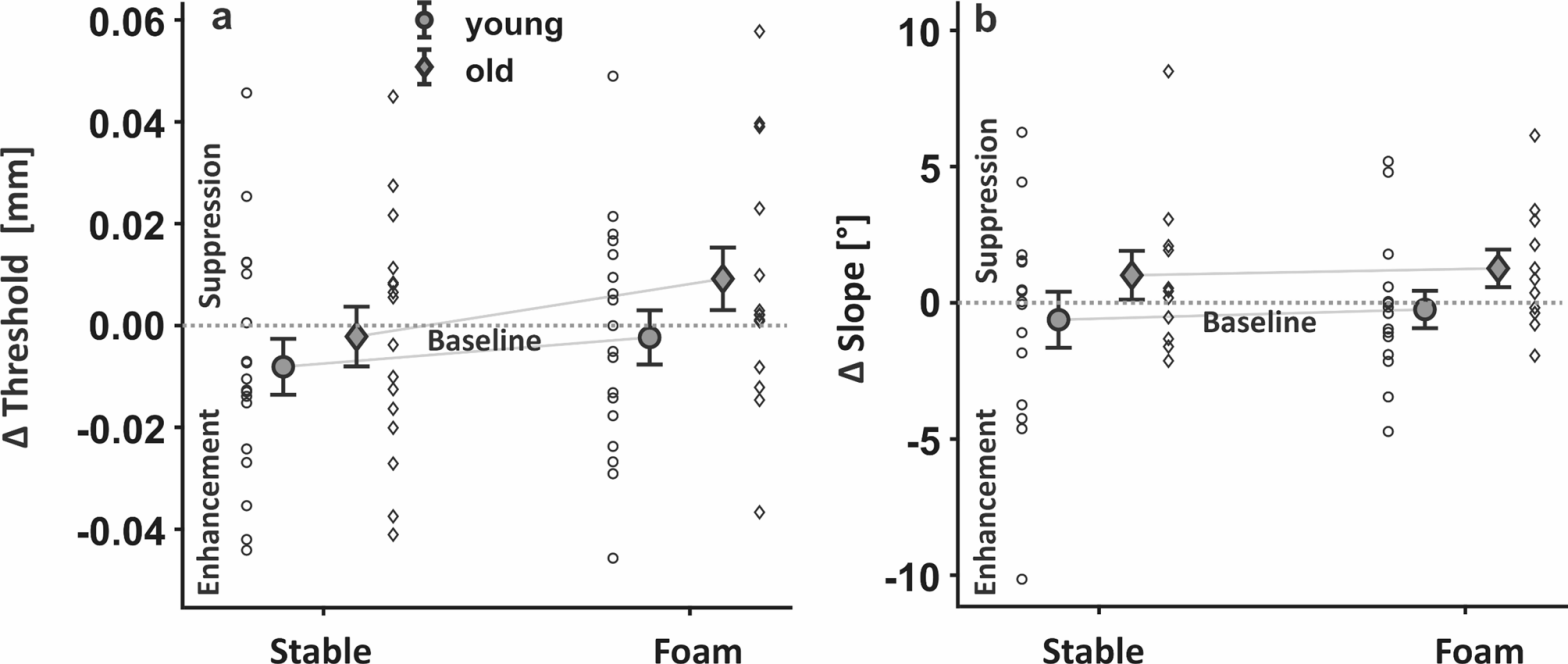
**Effects of age and postural demands on tactile modulation.** Comparison between postural demands and age group for (**a**) normalized detection thresholds, and (**b**) normalized slopes. Means and single subject data for the young (circles) and older adults (diamonds) are depicted with standard error as error bars. Horizontal dotted lines represent values at sitting (baseline).

After establishing that standing on foam influences posture and that aging reduces tactile sensitivity, we sought to examine how standing on stable and unstable surfaces influences tactile processing from the lower limbs in our two age groups. Tactile thresholds were higher when standing on foam than on the stable surface (*F*_*1,32*_ = 13.284, *p* < 0.001, *η*_*part*_^*2*^ = 0.293). However, there was neither a main effect of age *(F*_*1,32*_ = 1.282, *p* = 0.266, *η*_*part*_^*2*^ = 0.039), nor an interaction (*F*_*1,32*_ = 1.414, *p* = 0.243, *η*_*part*_^*2*^ = 0.042; Fig. 5a). There were also no main effects or an interaction for the slopes *(F*_*1,24*_ < = 2.508, *p* > = 0.126, *η*_*part*_^*2*^ *< =* 0.095; Fig. 5b).

## Discussion

We examined whether perception of tactile signals from the lower limb is modulated by postural demands and during healthy aging. We confirm previous results e.g.^43,44^, showing that standing on a surface of low compliance (foam) increases postural demands as reflected in pronounced postural sway. As expected^24,25^, we found poorer tactile sensitivity during sitting in older than in young adults. Our main finding is that tactile perception decreased with higher postural demands, and this decrease did not differ between the two age groups. Specifically, tactile sensitivity was poorer when standing on an unstable surface than on a stable surface in both young and older adults.

It may seem paradoxical that tactile sensitivity decreased when postural demands increased, because reduced tactile sensitivity from the lower limb may impede feedback processing that is relevant for the task at hand. Our results are in line with previous work showing poorer tactile perception when standing on a narrow base of support compared to sitting^21^. However, they are at odds with previous reports demonstrating improved tactile sensitivity on a limb that is involved in a task that requires pronounced sensory guidance during movement execution^34,45^, and around postural transitions^22,23^. Why did our participants perceive and incorporate less tactile information when their upright posture was challenged? One possible explanation comes from recent findings showing an increase in tactile suppression with an increase in global task load^46,47^. However, this possibility would not explain the above-mentioned results e.g.^22,45^, which found *weaker* tactile suppression or even tactile enhancement when task demands increase. A reduction in tactile perception when standing on foam than on a stable surface might indicate a shift in prioritization of posture at the expense of the simultaneously performed perceptual task e.g.^41^. However, the slopes of the psychometric functions did not differ between conditions, indicating similar performance in the discrimination of adjacent stimulus levels. Moreover, no changes in false alarm rates were observed across conditions or age groups, showing that response biases did not change. These two factors would suggest no change in prioritization; however, they cannot rule out this possibility entirely.

Another possibility that would explain the decreased sensitivity when standing on foam is that afferent signals from the leg, such as those arising from increased muscular activity to maintain upright stance, were stronger when standing on foam than on the stable surface. For instance, increased muscle activity or larger sway movements when standing on foam might have masked the tactile probe that we used to assess tactile sensitivity. Previous studies show that afferent signals can backward mask brief tactile probes presented around one’s own movement, and that such masking can partly explain tactile suppression^17^. This explanation also aligns with our previous study showing poorer tactile sensitivity when standing with a narrow base of support on a firm surface, which required increased muscular activations to maintain upright stance in a virtual reality setting^21^. However, in the current study we did not find tactile suppression when standing on the firm surface relative to sitting (modulation around zero in Fig. 5a; old: both *t* < = 1.796, both *p* > = 0.091, both *η*^*2*^ < = 0.045, young: both *t* < = 1.689, both *p* > = 0.109, both *η*^*2*^ < = 0.036). This might be due to some methodological differences, such as the vision of a virtual world environment versus real world, or differences in postural configuration. For instance, standing with the feet closed together, as done in the previous study^21^, limits the base of support and likely requires stronger muscle activations of the calf, compared to when standing with a wide base of support, as done in the current study. The possibility of increased muscular activations when standing on a narrow base of support might have led to pronounced masking processes that could have impaired perception of the probing stimuli in the previous study. However, considering that the influence of afferent signals on tactile masking is not systematic e.g.^16,48^, we can only speculate about the precise mechanisms behind the decreased tactile sensitivity with higher postural demand.

Although aging led to higher detection thresholds during sitting, confirming that aging compromised tactile sensitivity, we did *not* find any evidence that postural demands influence tactile sensitivity differently in young and older adults. This lack of an age effect may be explained by the absence of age-related differences in sway area or path length, which could have indicated a greater need for modulation of tactile processing in older adults. The lack of an age-effect in sway area is in line with previous findings showing that postural sway does not differ between older and younger adults^49^ or it may be even smaller in aging^50^. Older adults can compensate for their sensorimotor decline by increasing the co-activation of their ankle muscles^51^. In this case, one might have expected stronger masking of the probing stimulus due to the muscle stiffness, which we do not observe. Please note that we have not measured muscular activity, so this remains a hypothesis for future research. In sum, we show that tactile suppression during standing is stronger with higher postural demands in both young and older adults. These results highlight the contribution of tactile information in postural control and their dependencies on the task demands irrespective of age.

### Limitations

The presented work is primarily limited by two factors. First, our cohort of older adults is relatively young, given that we included participants above the age of 55 years. However, as sensory decline is a gradual process, a 40-year difference between the two groups, as evident in our samples, enables us to examine how sensorimotor processes, such as tactile sensitivity and postural control, are reflected in the aging sensorimotor system. The observed effects may become even more pronounced with increasing age, as sensory and motor processes are further declined^26^.

The second limitation pertains to the relatively short trial durations employed in the kinetic analysis. Although this duration allows for the assessment of changes in postural control, it limits the accurate quantification of lower-frequency sway components (below 0.5 Hz), which are known to contribute substantially to overall postural sway^52^. Precise estimation of these frequencies would require longer trial durations. However, this was not feasible in our psychophysical study with the primary focus on sensory modulations, which necessitates a high number of trials for reliable sensory threshold quantification. Nonetheless, we were able to demonstrate kinetic changes associated with postural demands; thus, short trial durations are sufficient to capture the increased postural sway when standing on surfaces of lower compliance. The lack of an age-effect on postural behavior may come as a surprise, however, it is consistent with previous studies that did not obtain evidence for age-related differences in balance control, even when using longer trial durations^49^.

## Data Availability

Behavioral and psychophysical data are publicly available after acceptance at https://osf.io/jwghy/. For further information or data requests please correspond to Fabian Dominik Wachsmann (Fabian.Wachsmann@psychol.uni-giessen.de).
